# Ebstein’s anomaly: an electrophysiological perspective

**DOI:** 10.1007/s10840-024-01744-8

**Published:** 2024-01-30

**Authors:** Anunay Gupta, Mukund A. Prabhu, Robert D. Anderson, Srinivas BV. Prasad, Timothy Campbell, Samual Turnbull, Geoffrey Lee, Jonathan R. Skinner, Jonathan Kalman, Saurabh Kumar

**Affiliations:** 1 Department of Cardiology, Westmead Hospital, Westmead Applied Research Centre, University of Sydney, Darcy Road, Westmead, Sydney, New South Wales 2145 Australia; 2 Department of Cardiology, Vardhman Mahavir Medical College and Safdarjung Hospital, New Delhi, India; 3 Department of Cardiology, Kasturba Medical College Manipal, Manipal Academy of Higher Education, Manipal, Karnataka India; 4 Department of Cardiology, Royal Melbourne Hospital, University of Melbourne, Parkville, Victoria Australia; 5 Department of Cardiology, Fortis Hospital, Bannerghatta, Bengaluru India; 6 Department of Cardiology, Children’s Hospital Westmead, Westmead, New South Wales Australia

**Keywords:** Ebstein anomaly, Arrhythmia, Ablation

## Abstract

Ebstein’s anomaly of the tricuspid valve (EA) is an uncommon congenital cardiac malformation. It can present with atrioventricular tachycardia (AVRT), atrioventricular nodal re-entrant tachycardia (AVNRT), atrial arrhythmias, and rarely with ventricular tachycardia. The 12-lead electrocardiogram (ECG) is critically important and often diagnostic even prior to an electrophysiology study (EPS). Due to its complex anatomy, it poses particular challenges for mapping and ablation, even for an experienced electrophysiologist. In this review, we aim to provide insight into the electrophysiological perspective of EA and an in-depth analysis of the various arrhythmias encountered in diverse clinical scenarios.

**Graphical Abstract:**

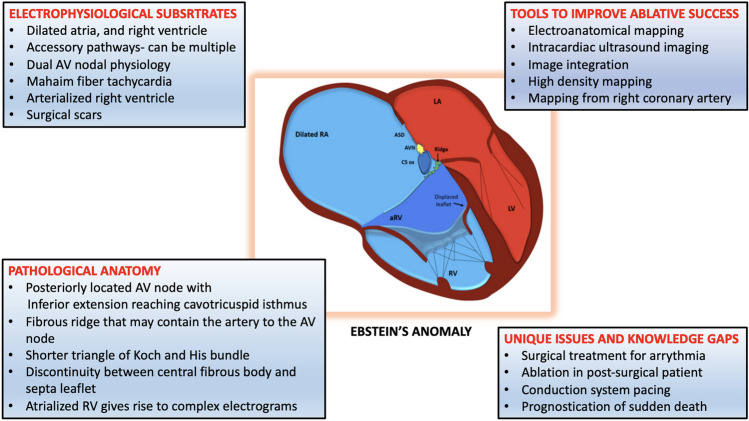

## Introduction

Ebstein anomaly (EA) accounts for < 1% of congenital heart disease. The incidence of accessory atrioventricular (AV) connections is distinctly higher in EA (10–23%) [1]. However, atrioventricular nodal re-entrant arrhythmias, atrial arrhythmias, and, more rarely, ventricular arrhythmias can be seen too. The 12-lead electrocardiogram (ECG) is critically important and often diagnostic even prior to an electrophysiology study (EPS) [2]. Further, the anatomical abnormalities seen in EA can pose unique challenges to the electrophysiologist during invasive EPS. This review delves into a comprehensive exploration of the anatomical characteristics, various encountered arrhythmias, and their management.

## Anatomical considerations

**Fig. 1:**
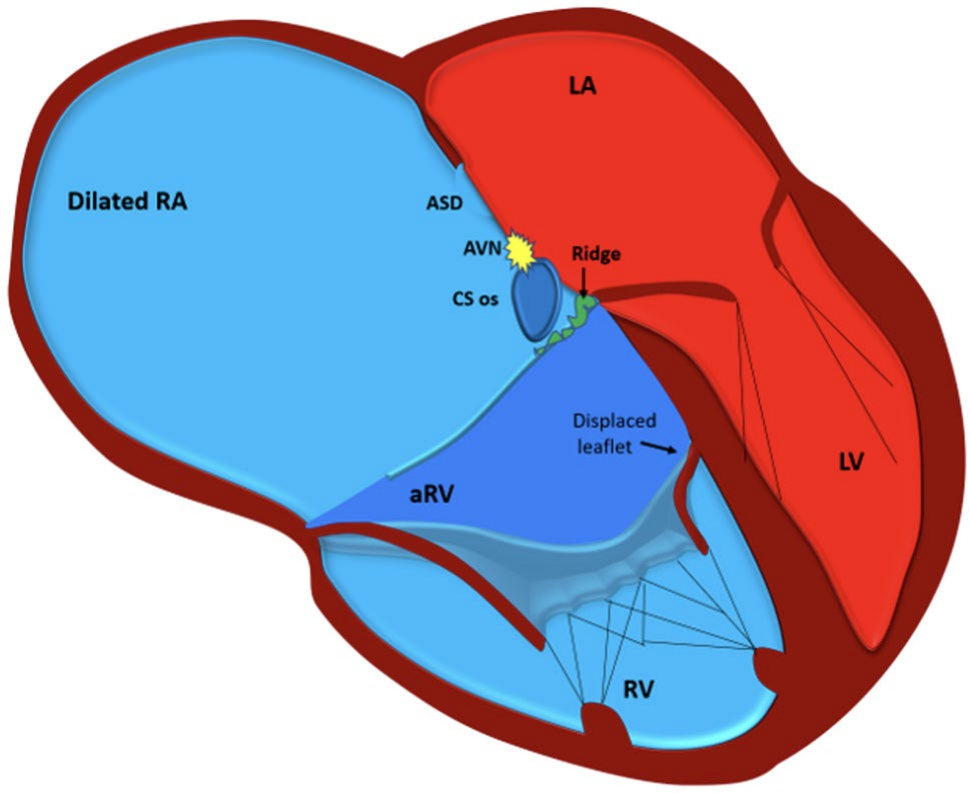
Illustration of a heart with Ebstein’s anomaly highlighting the anatomical abnormalities relevant to the electrophysiologist. The right atrium (RA) is dilated and so is the tricuspid annulus. The septal tricuspid leaflet is apically displaced, and there is a fibromuscular ridge instead (arrow) which forms the boundary of the triangle of Koch which is smaller compared to normal hearts. The coronary sinus (CS) ostium is dilated, and the AV node (AVN) is irregular, and its body can reach the upper border of the CS ostium unlike in normal hearts where only its inferior extension reaches here. ASD, atrial septal defect; aRV, atrialised RV; LA, left atrium; LV, left ventricle; RV, right ventricle

The hallmark of EA is malformation of the tricuspid valve (TV), with apical displacement of the insertion of its septal leaflet (Fig. 1). Abnormalities seen in EA include adherence of the septal and posterior TV leaflets of the underlying myocardium, a “sail-like” anterior leaflet of the TV with redundancy and tethering, apical displacement of the functional tricuspid annulus resulting in an atrialised portion of the right ventricle (ARV), and dilation of the right AV junction and the right atrium (RA) [3]. The ARV is the inlet portion of the right ventricle (RV) which is thin and continuous with the RA, whereas the functional RV constitutes of the trabecular and the outflow portions can vary in size depending on the severity of EA [4, 5]. The large anterior tricuspid leaflet (ATL) may cause difficulty manoeuvring the catheter and attaining a good annular contact from the ventricular aspect.

### Pathological anatomy for the electrophysiologist

The RA can be grossly dilated in EA [6]. The ARV is thinned and deficient in myocytes and can be aneurysmal and dyskinetic. The functional RV too has varying degrees of fibrosis which may also involve the left ventricle as well in severe cases of EA [6]. These substrate changes create the relevant electroanatomical substrate for re-entrant atrial and ventricular arrhythmias in EA.

Studies on foetal and perinatal hearts have showed that the triangle of Koch (KT) is smaller in the hearts with EA compared to normal hearts [7, 8]. A larger coronary sinus (CS) ostium and the presence of an endocardial fibromuscular ridge that marks the anterior extend of KT are important anatomical variations to be cognisant of, since up to 25% of the EA patients, the artery to the atrioventricular node (AVN) runs through this ridge. The body of the AVN is shifted downwards to the base of the KT, and the inferior extensions reach the level of the cavotricuspid isthmus (CTI) even in perinatal hearts [7]. The His bundle (HB) is found more commonly to begin before the apex of KT and is significantly shorter in length in the hearts with EA than in normal hearts [7]. While the KT is smaller, the actual length of the AVN is similar to that in normal hearts in EA which means that the AVN occupies the even more of the KT space. This also distorts the shape of the AVN which is more variable. The muscular AV septum which is inclined in normal hearts is more horizontal in EA as the annular rings insert at the same level into the central fibrous body (CFB) [9]. The apical displacement of the STL results in discontinuity between the CFB and the septal AV ring, thus creating the substrate for accessory AV connections [7]. A prominent ridge along the inferior right atrioventricular groove is present, and it may make ablation difficult in patients with accessory pathways [9].

## The electrocardiogram (ECG)

**Fig. 2:**
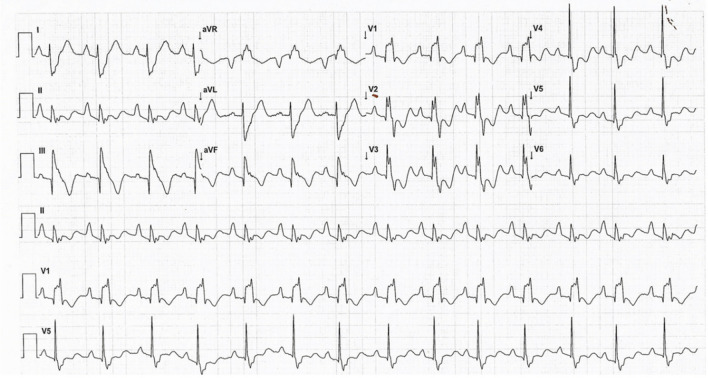
The standard 12-lead electrocardiogram in an 11-year-old boy with severe Ebstein’s anomaly demonstrating peaked P waves, prolonged PR interval, and a splintered QRS complex. The leads V1 and aVR have similar morphologies of the QRS complex (see text for details)

The ECG may show distinct features reflecting the structural changes seen in severe EA (Fig. 2). The P wave shows right atrial (RA) enlargement in up to 75% cases [10–12]. The PR interval is prolonged in 15% patients, but complete AV block is rare [11]. The PR prolongation is due to the delay in atrial rather than the AV nodal conduction and explains the occurrence of a normal PR interval even with preexcitation.

**Fig. 3:**
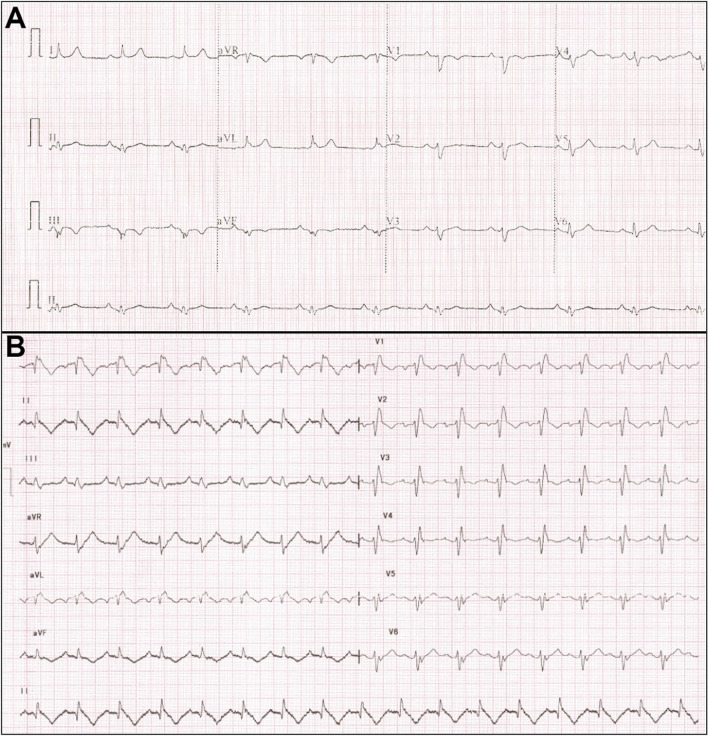
12-lead ECG in a patient with Ebstein anomaly showing normal PR interval and no delta waves with the absence of RBBB in lead V1 (**A**). This is suggestive of right-sided pathway. Patient underwent successful ablation of the right posterior bypass tract, and the postablation ECG showed typical RBBB pattern in lead V1 (**B**)

The QRS complex in EA is typically abnormal. Varying degrees of right bundle branch block (RBBB) pattern has been reported in 75–92% cases of EA and may be due to the abnormal development of the septal leaflet and medial papillary muscle [13]. The absence of a RBBB pattern at baseline has a 91% predictive value for the diagnosis of a right-sided accessory pathway in a patient with EA and supraventricular tachycardia (SVT) [14] (Fig. 3). Apart from RBBB, the QRS complex may show various other changes such as a deep q wave in V1, or in V1–V3 (possibly due to fibrotic areas in the septum), deep q waves in the inferior leads, and delayed r′/R′ upstrokes in the inferior leads due to the late activation of the ARV [15, 16]. The QRS may appear as a splintered polyphasic morphology in EA due to the late abnormal conduction through the ARV. The dilated RA tends to cause the precordial leads V1–V3 to record intracavitary right ventricular (RV) potentials resulting in the q waves in these leads and V1 morphologically mimicking the lead aVR in severe cases of EA [15].

## Arrhythmias

Ebstein’s anomaly harbours substrate for the entire spectra of atrial and ventricular arrhythmias. Supraventricular arrhythmias are common and include atrial tachycardia, atrial flutter, atrial fibrillation, and AV node–dependent re-entrant tachycardias. Accessory pathways are found more often in patients with EA and cause atrioventricular re-entrant tachycardias (AVRT). Though less often than AVRT, atrioventricular nodal re-entrant tachycardia (AVNRT) occurs in EA. Both atrial and ventricular premature beats are reported to occur in EA. The atrialised RV forms the classic substrate for ventricular tachycardia (VT). Bradyarrhythmias are less common, and the first-degree AV block commonly seen is predominantly due to the prolonged P wave duration rather than AV nodal involvement. Nearly a third of the patients have more than one arrhythmia mechanism, and one-fifth may require multiple ablative procedures for cure [17]. Table 1 summarises the outcomes of arrhythmia ablations in EA.

**Table 1:** The outcomes of arrhythmia ablations in Ebstein anomaly

Study	Design	Population	Number of patients	Arrhythmias	Follow-up	Outcomes
Reich et al. (2007)	Retrospective (26 centres)	Paediatric EA, mean age 9.85 ± 4 years	65	82 typical AP, 17 other SVT (1 FAT, 7 AVNRT, 5 Mahaim, 4 IART), and 1 VT	NA	EA acute success rates and recurrence rates for right free wall, right septal, and other mechanisms were 79%/32%, 89%/29%, and 75%/27%, respectively
Roten et al. (2011)	Retrospective (4 centres)	Patients with SVT referred for catheter ablation Mean age 24 ± 15 years	32	34 AP, 8 IART, 5 CTI-AFL, 2 FAT, and 1 AVNRT	NA	Acute procedural success rate—80% for AP/CTI-AFL and 100% for IART AP—recurrence rate 40% CTI-AFl—recurrence rate 60%
Wei et al. (2014)	Retrospective (single centre)	Adults with accessory pathways undergoing EPS	17	24 AVRT with 23 AP 20 (87%)—nondecremental bypass tract 2 (8.7%)—decremental bypass tract 1 (4.3%)—nodofascicular tract	11.9 ± 6.8 months	No recurrence
Orczykowski et al. (2017)	Retrospective (single centre)	Adults with accessory pathways undergoing RFA	22	33 AP, 8 (36%) multiple APs, 20 AVRT (orthodromic), 5 AVRT (antidromic), 3 AVNRT, 1 AFL (CTI)	95.7 ± 49.8 months	Acute and long-term success rate—100%/100% for AVNRT, 77.3%/95.5% for APs, and 50.0%/100% for CTI-AFL ablation
Wackel et al. (2018)	Retrospective (single centre)	Age < 21 years undergoing cone repair Median age, 10 years (0.1–20.9 yrs.)	143 35 (24%)—preop EPS 108 (76%)—no preop EPS	26 (18%) had preoperative ablation, 9 (6%)—negative EPS. 33 arrhythmias targeted AP (25), AVNRT (5), VT (1), FAT (2)	2.9 years (0.1–9.2 years)	3 patients in both no preop EPS and preop EPS had ablations on follow-up
Hassan et al. (2018)	Retrospective (single centre)	64 (12–71), patients with atrial arrhythmias	22, all with history of cardiac surgery	Atrial flutter (63.6%) FAT (22.7%) Atrial fibrillation (9.1%)	NA	1-year recurrence rate—10% 5-year recurrence rate—41.2%
Moore et al. (2018)	Retrospective (11 centres)	Post-surgical/unoperated Median age 17 yrs. (11–37)	24 12—post-surgical 12—unoperated	28 VA substrates, 25 completely characterised. 15 were focal and 10 were re-entrant	3.4 years	92% success rate
Moore et al. (2019)	Retrospective (7 centres)	Post-surgical/unoperated	42—TV ring/replacement 39—TV repair 55—no TV surgery	CTI-dependent IART (36%) IART (29%), FAT (18%), SVT (17%)	3 years	26% recurrence rate
Asaad et al. (2021)	Retrospective (single centre)	Age 9 (2.6–13.3) with accessory pathways	76	52 had AP, 9 had AP plus additional arrhythmia	2.5 (0.2–7 years)	Multiple procedures: success rate—93%. 1-year recurrence rate—19%
Karagoz et al. (2022)	Retrospective (single centre)	All patients with diagnosis of EA Median age 14 (range, 4–17) years	26	33 procedures (including recurrence) AP (27), AFl (5), AVNRT (5), AFl (2)	6.26 (range, 1–17) years	AP: 1-year recurrence rate—15%

*AP* accessory pathway, *AF* atrial fibrillation, *AFL* atrial flutter, *IART* intraatrial reentrant tachycardia, *AVNRT* atrioventricular nodal reentrant tachycardia, *AVRT* atrioventricular reentrant tachycardia, *CTI* cavotricuspid isthmus, *FAT* focal atrial tachycardia, *EPS* electrophysiological study, *SVT* supraventricular tachycardia, *VT* ventricular tachycardia

### Electrophysiological study

**Fig. 4:**
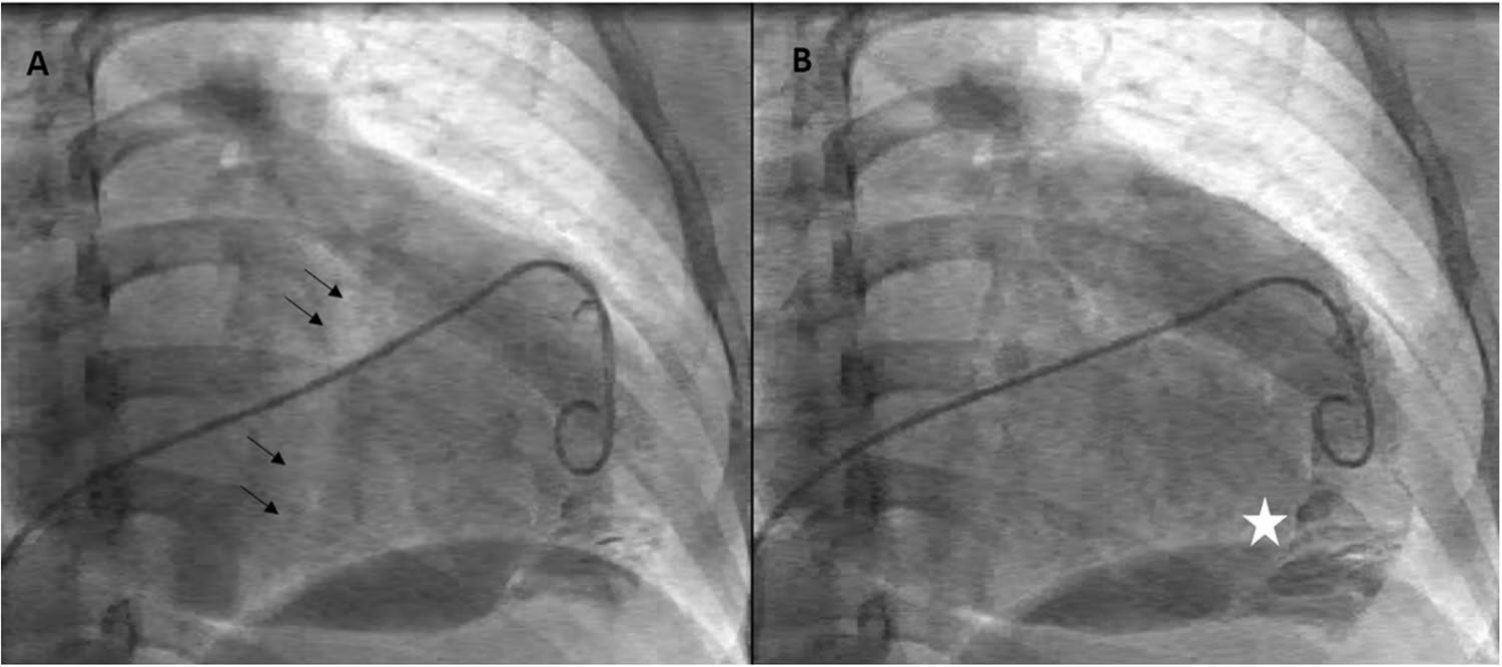
Still frames from a right ventriculogram in the right anterior oblique 20 degree view in a patient with Ebstein’s anomaly. The black arrows (**A**) mark the atrioventricular groove whereas the white asterisk (**B**) marks the apically displaced tricuspid valve leaflet

**Fig. 5:**
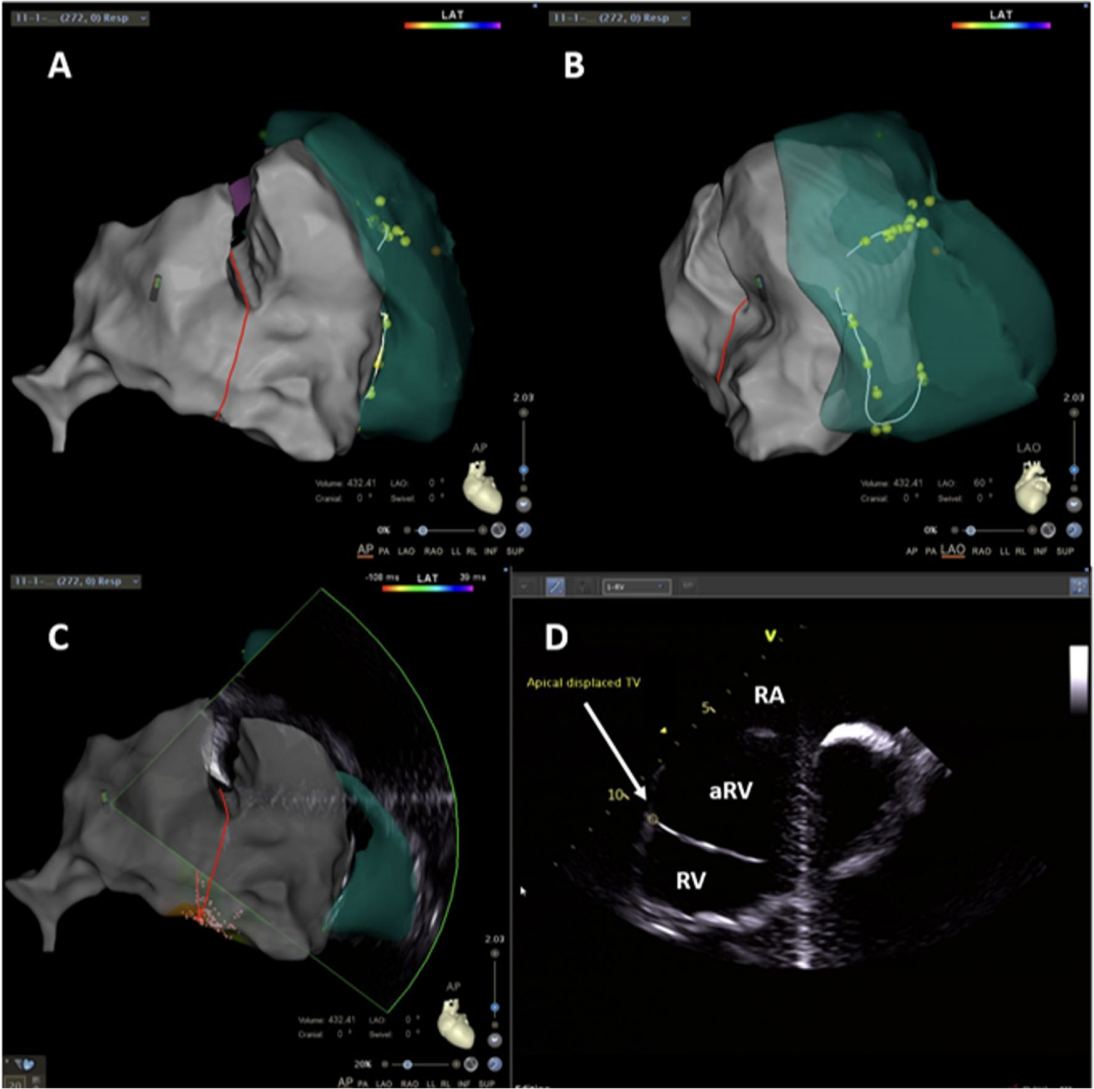
Images using CARTO system (Biosense Webster) with intracardiac echocardiography. A Vision Wire was placed in the right coronary artery to mark the plane of the true annulus. Panels **A** and **B** represent the AP and the LAO views, respectively. Panel **C** shows the plane of the ultrasound beam used to derive the image in the panel **D**. The red line denotes the plane of the tricuspid annulus, while the yellow tags and the connecting white line marks the plane of the displaced tricuspid leaflet (arrow in **D**). Also marked are the right atrium (RA), the atrialised right ventricle (aRV), and the right ventricle (RV)

**Fig. 6:**
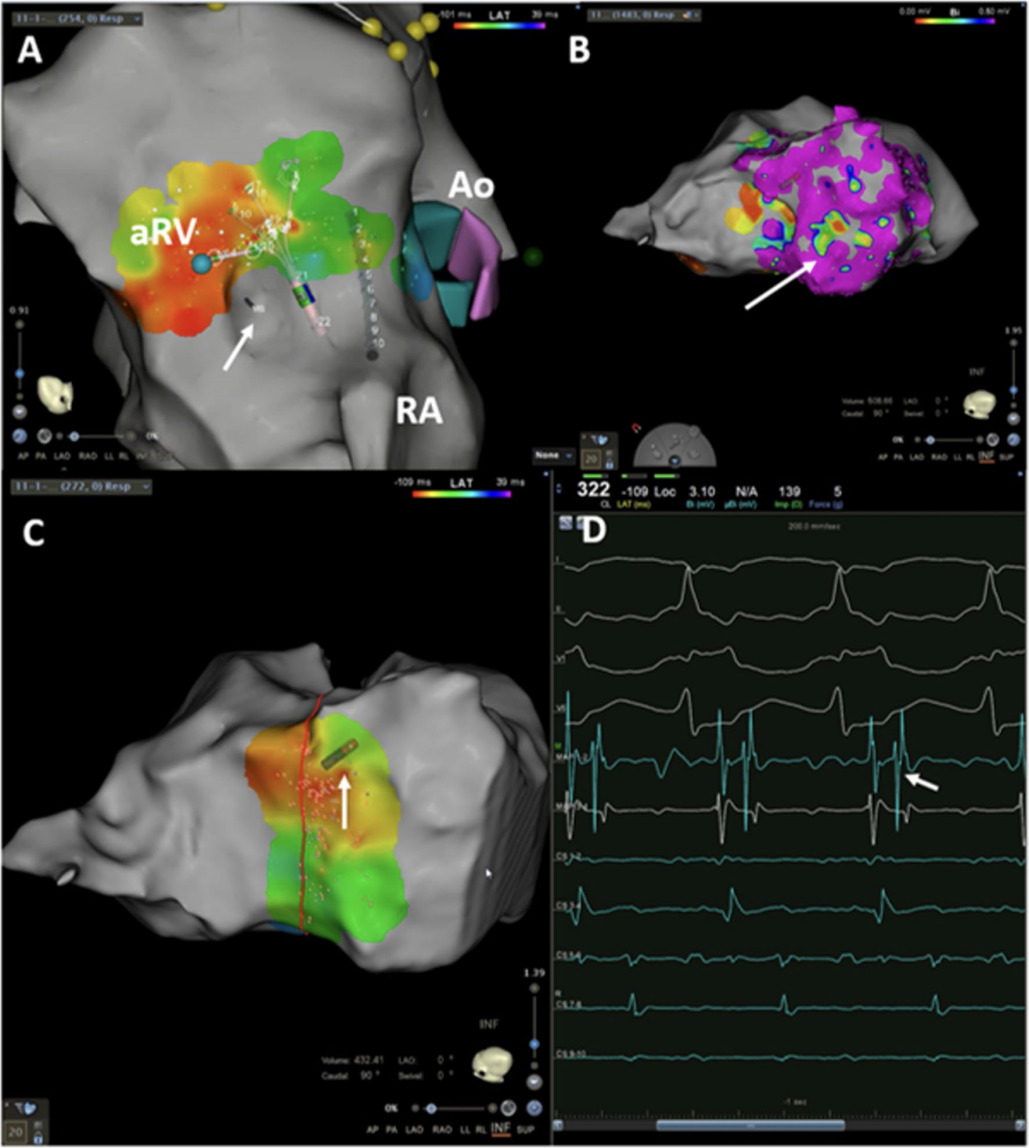
**A** The activation map with a PentaRay high-density mapping catheter during an orthodromic AVRT showing the earliest activation in the aRV. A 0.014-inch Vision Wire guidewire (Biotronik SE and Co. KG, Berlin, Germany) is seen floating adjacent and posterior to the annulus. Also marked is the aortic root (Ao) and the RA. Panel **B** shows the voltage map of the aRV with some low voltage areas (arrow). Panel **C** shows the successful site of ablation in the posterior annulus, and the corresponding signals are shown in the panel **D**. Note that the atrial signal (arrow), though the earliest, is not fused with the aRV signal perhaps due to a slow conducting pathway

Identifying the tricuspid annular plane in the fluoroscopic right anterior oblique (RAO) view is critical since the annular electrograms (EGMs) can be confusing in EA due to the electrograms (EGMs) recorded from the RA(A), aRV, and the RV(V) giving rise to three distinct EGMs. The RAO 20° view is a preferred starting point, and the radiolucent fat pad marks the annular plane (Fig. 4). Improved delineation can be achieved by a right coronary angiography and can provide a reasonable reference [18]. Three-dimensional electroanatomical mapping can be used as a good anatomical guide especially when merged with intracardiac ultrasound visualisation (Fig. 5). The use of high-density catheter (PentaRay, Biosense, or HD-Grid, Abbott) along with the open-window annotation algorithm (Abbott’s NavX Precision or Biosense Webster Carto3) can improve the success rate in redo cases (Fig. 6). Intracardiac echo or preoperative imaging (CT or MRI) may also be used for image integration. Using intracardiac echo, one may be able to differentiate the right atrium, aRV, RV, and the valve annulus for accurate mapping and ablation.

**Fig. 7:**
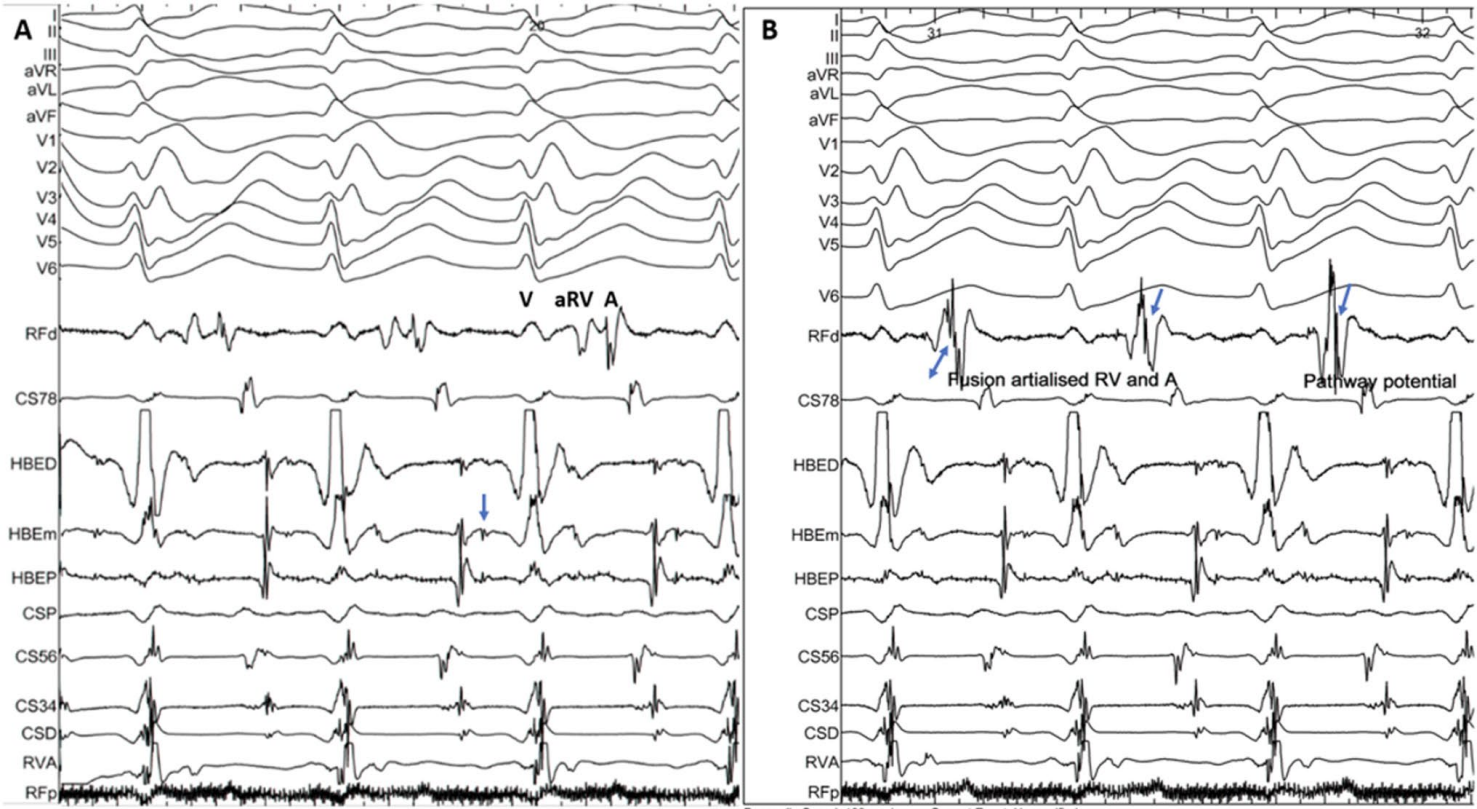
Electrograms during an orthodromic AVRT in a patient with Ebstein’s anomaly. Panel **A** shows the signals in the mapping catheter as it maps the tricuspid annulus. The EGMs representing ventricle, the atrialised RV, and the atria are marked V, aRV, and A, respectively. The arrow marks the His potential preceding the ventricular EGM in the His catheter. In panel **B**, the mapping catheter is positioned at the site of successful ablation at the posteroseptal tricuspid annulus. The EGMs from the aRV and the A are fused (double-headed arrow), and the pathway potential is seen (arrows). In both the panels, RFd and RFp represent the distal and proximal bipoles of the mapping catheter. HBd, HBm, and HBp represents the distal, mid, and proximal bipoles of the hexapolar His bundle catheter. CSp to CSd represent the proximal and distal bipoles of the coronary sinus decapolar catheter with the other bipoles sequentially numbered. The RFd and CS78 have been displayed adjacent to the surface electrocardiogram leads to make it easier for timing the ventricular and the atrial EGMs

The annular EGM can be complex and fractionated, especially in the ventricular aspect (Fig. 7). Differentiating the aRV signal from the RA signal is an important initial step. During sinus rhythm, this can be achieved by extra stimulus protocol being delivered from the atria until atrioventricular refractoriness when a “pure” atrial EGM can be appreciated. Similarly, pacing from the RV till ventriculoatrial refractoriness will dissociate the aRV-V EGM complex from the A. This will confirm that the catheter is at the atrial aspect of the annulus or the aRV and avoid inadvertent ablation. Importantly, the presence of such complex EGMs cannot be predicted from the extent of the apical displacement of the TV, the area of the aRV, or the septal thickness and may be complex even in relatively mild forms of EA.

APs can be easily “bumped” during catheter manipulation, and subsequent temporary lack of conduction may necessitate study abandonment. Peri-procedural anticoagulation requires careful planning in the presence of an atrial septal defect and tricuspid regurgitation to avoid paradoxical embolism.

The PA and HV interval may be prolonged reflecting conduction delay in the dilated right atrium. The VA interval can be prolonged in some of the patients associated with orthodromic AVRT with a long RP tachycardia which may confuse the diagnosis. This delay in the retrograde conduction might be due to the delay in activation of the aRV and the adjacent atrium [19].

### Atrial arrhythmias

Atrial arrhythmias (AA) are common in adults with EA. In 682 adults with mean age of 36 years, the prevalence of AA at baseline was 34% (21% had AFl and 20% had AF). In patients who do not have AA at baseline, 10-year cumulative incidence of AF and AFL was 16% and 22%, respectively [20]. Male gender, older age, and atrial septal defects were predictors of AA at baseline [20]. Most atrial arrhythmia circuits in EA are confined to the right atrium; the cavotricuspid isthmus (CTI)-dependent flutter is the commonest [21]. However, the CTI may be distorted due to the displacement of the valve leaflets and RA dilation. Despite this, ablation has reasonable success in curing flutter [21].

Atrial tachycardia (AT) is associated with poor outcomes. In many cases, it precedes the development of ventricular tachycardia and death. The occurrence of AT correlated with derangement of the right heart parameters in cardiac magnetic resonance imaging (CMR) [22]. Atrial fibrillation is an increasing issue as patients survive for a longer age, and there are reports of pulmonary vein isolation with reasonable medium term results [23]. However, in patients undergoing surgical repair for EA and who have AF, a MAZE procedure during the surgery is the preferred strategy [24].

### AVNRT

About 10% of patients with EA and SVT have AVNRT which may coexist with APs or Mahaim fibres [25]. A recent study observes that congenital heart disease with right atrial pressure or volume overload has a predilection to develop AVNRT, presumably because of the hemodynamic effects causing fibrosis, anisotropic conduction, and thus slow pathway conduction [26]. The anatomical abnormality of the KT has previously been discussed, but it should be emphasised that care should be taken to prevent inadvertent ablation of the posteriorly displaced AVN. The electrograms from the aRV can be of low voltage and thus create confusion while targeting successful sites for potential ablation by the EGM characteristics. Both conventional radiofrequency ablation and perinodal surgical cryoablation have satisfactory outcomes in eliminating the AVNRT [25].

### AVRT

Though EA form a small subset of patients undergoing ablation for APs, the incidence of APs occurring in EA is much higher and incidence is reported between in up to 1/3rd of patients depending upon the study population and whether pathways were concealed or manifest [27]. Moreover, almost always these pathways are confined to the tricuspid annulus and often multiple APs coexist. In the absence of AV discordance, 98–100% of the APs are right sided along the TA [25, 28]. Within the TA, the posteroseptal and posterior regions are the most common regions to harbour APs [17, 29]. The APs may be concealed and may not be manifested on the ECG alone, highlighting the need of EPS in symptomatic patients with palpitations and in those planned for surgical repair. The APs are similar to those occurring in structurally normal heart with respect to their effective refractory period and the shortest R-R interval during AF [30, 31]. However, the occurrence of multiple APs ranging from 15 to 34% is disproportionately higher in EA than structurally normal hearts [29, 32, 33].

Many factors adversely affect the successful ablation of APs in EA and risk of recurrence. The complex signals at the TA, presence of multiple pathways, the dilated RA, and distorted annular geometry restricting catheter contact are all contributors. Hence, the risk of recurrence is relatively high (20 to 40%). One study reported that the risk of recurrence of an AP after ablation at 1 year was 19% in children aged 2.6 to 13.3 years [34]. In a retrospective case series of 39 paediatric patients undergoing redo ablation, 4 (10%) had EA [35]. In normal hearts, a V > A ratio is considered desirable at the target site of ablation. In contrast, in EA with fractionated EGMs, a higher A/V ratio may guide the successful site than when fractionated EGMs are absent. A cutoff of > 0.6 has been proposed when there is EGM fractionation as opposed to a normal EGM, a ratio of < 0.6 is accepted [36]. The CT and MRI images may be difficult to merge for the lack of standard reference points. The voltage map may also reveal low voltage scar areas within the aRV. The APs may be broad and result in wide regions of early activation contributing to the complexity in mapping [29].

### Mahaim tachycardia

The incidence of Mahaim fibres in case series of ablation of AP Ebstein anomaly is around 7–8%—much higher than the general population [28]. Mahaim fibres are atriofascicular accessory pathways with decremental conduction properties. In general, they conduct only in the antegrade direction and hence participate antidromic tachycardia. The antidromic tachycardia has a left bundle branch block (LBBB) pattern of the QRS complex and leftward axis shift in the frontal plane. The RP interval is usually short, reflecting brisk retrograde conduction through the His-Purkinje system which also results in the right bundle branch (RBB) activation to precede the His potential during the tachycardia (RBB-His reversal). The electrophysiological hallmark of “Mahaim physiology” is progressive lengthening of the AH interval with shortening HV interval and increasing degree of preexcitation during atrial pacing at faster rates. The pathway is usually located in the lateral tricuspid annulus, and a distinct “M-potential” is classically seen while mapping this site and is also the target for successful ablation. The electrophysiological features, common sites, and ablative targets of Mahaim fibres are the same in patients with or without EA and have been elegantly reviewed elsewhere [37]. However, the dilated right atrium and the large tricuspid leaflet may pose difficulty in attaining catheter stability and contact in patients with EA, and a long sheath like the Schwartz right (SR), SR3, or the deflectable Agilis sheath (Abbott Medical, Chicago, Illinois, USA) is commonly needed. To add complexity to delineating arrhythmia mechanism, atriofascicular APs frequently coexist with typical APs [25, 29]. Wei et al. reported success rate of 100% with decremental bypass tract and no recurrence at median follow-up of 11.9 months [29].

### Ventricular tachycardia

**Fig. 8:**
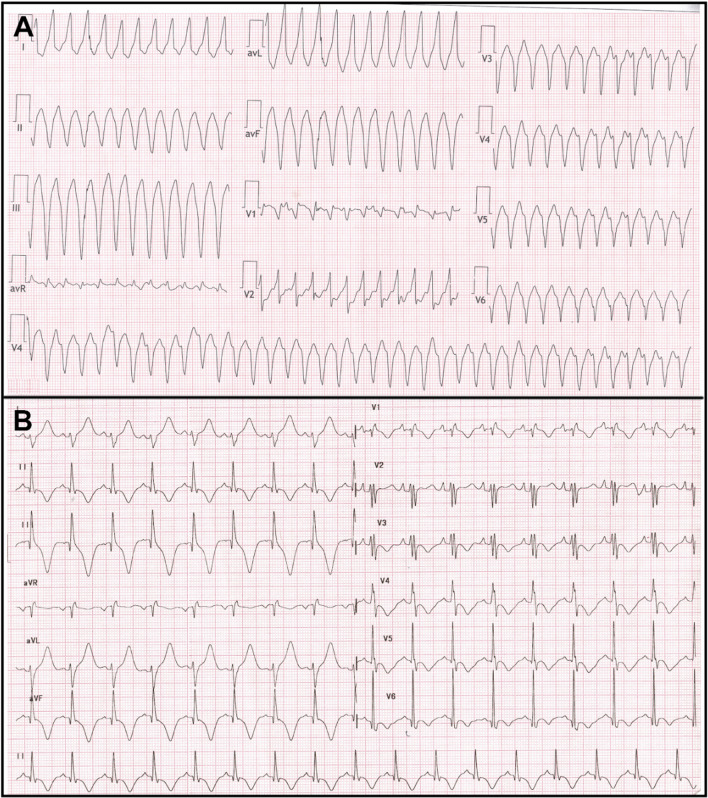
**A** Twelve lead ECG of a young adult with ventricular tachycardia which was successfully ablated from atrialised right ventricle. **B** The baseline ECG after tachycardia termination showed presence of RBBB in lead V1 and memory T waves in inferolateral leads. No SVT was induced during EP study

Ventricular tachycardia is the least common tachycardia seen but is being increasingly reported. Recently, a multicentre study by Moore et al. summarised the experience of ventricular tachycardia (VT) ablation in 24 patients undergoing VT ablation at 11 centres [38]. Twelve patients (50%) have undergone tricuspid valve surgery. VA mechanisms were focal in 15 and macro-re-entrant in 10 and did not differ significantly between those with and those without prior TV surgery. Focal VAs predominantly localised to the ARV in unoperated patients and to diseased myocardium or Purkinje tissue after TV surgery. Macro-re-entry was seen in ARV in unoperated patients and from scar after TV surgery. Complete success was achieved in 22 (92%) [38]. Figure 8 shows a 12-lead ECG of young adult who presented with VT and was successfully ablated from atrialised RV. Rydman et al. observed that cardiac MRI–derived parameters of ventricular dysfunction were associated with the development of ventricular tachycardia and death [22]. The aRV has areas of fibrosis interspersed with myocardial tissue that predisposes scar re-entry. Linear ablation lines between the true annulus and the adjacent scar is the preferred ablation approach. This technique is not without potential risk, particularly given the thin-walled aRV, and there is a risk of perforation or dissection of the aRV [39].

## Additional technical considerations

### Right coronary mapping

The right coronary artery (RCA) lies in the anatomical AV groove and has been used by some for mapping for the AP if conventional mapping fails. However, the artery should be normal and sizeable enough and preferably extend up to the crux. Furthermore, this needs a specially designed 2F multi-electrode catheter with four electrodes spaced 3 mm apart (centre-to-centre) (Corotrax, Sulzer-Osypka, Grenzach-Whylen, Germany). The catheter is slowly withdrawn from the crux towards the proximal RCA concurrently mapping for the earliest antegrade V/retrograde A. The earliest site thus obtained acts as an anatomical guide for the endocardial site for successful ablation which should also have matching EGMs [40, 41]. Alternatively, a Vision Wire (Biotronik, Berlin, Germany) which is a specially coated 0.014 inch coronary guidewire positioned in the RCA can be used to mark the plane of true annulus (Fig. 6).

### Intracardiac echocardiography

Intracardiac echocardiography (ICE) is being increasingly utilised during ventricular arrhythmia ablation to characterise substrate. ICE can help in characterising true tricuspid annulus and facilitating the mapping efforts on the true AV ring instead of the atrialised RV. The arrhythmia recurrence rate was 0% after a mean follow-up of 16.16 ± 7.7 months in a case series of 6 EA patients with ICE-guided ablation in which 50% patients had past history of ablation [42].

## Surgical repair

The surgical repair in EA gives the opportunity for surgical modification the arrhythmia substrate but may also inadvertently create additional arrhythmia substrates related to the surgical incision. Given the preponderance of arrhythmias in EA, and with reports of sudden death in patients with EA after successful surgical repair, an electrophysiological study prior to the surgery is ideal even in the absence of arrhythmia symptoms [43, 44]. As such, current guidelines recommend preoperative EP study even in asymptomatic patients planned for surgical repair (class IIa, B-NR) [45].

### Surgical procedures for correction of hemodynamic abnormalities

The surgical procedure for correcting the TV abnormality in EA has significantly improved and evolved since first operated on over 50 years ago. In brief, the initial techniques involved the plication of the aRV aiming to reduce the annular size and improve the apposition of the leaflets including a Danielson’s repair. In some cases, the annuloplasty requires the use of a prosthetic ring, and less commonly, a TV replacement is required. While the details of these are beyond the scope of this review and are covered in other excellent reviews [46], it is pertinent for the electrophysiologist to have an understanding about tricuspid annuloplasty and TV replacement, since these procedures can alter the anatomy of the annular region relevant to ablation of arrhythmias like the CTI-dependent flutter or the VT arising from the aRV.

### Surgical procedures for arrhythmia control

The addition of surgical ablation is reserved for failed catheter-based ablation, or a recurrence after the successful one in a patient planned for surgical repair for EA in the current era. The addition of surgical ablation along with the repair confers no additional mortality risk. In general, all right-sided APs can be surgically ablated without cold cardioplegic arrest or hypothermia, as opposed to left-sided pathways. Ablation of the APs precedes the tricuspid valve surgery repair/replacement so that access to the annulus is easier [31].

### Arrhythmias in the post-surgical patient

Incision-related arrhythmias can be successfully ablated with RF ablation, but a CTI ablation can be difficult if a tricuspid annuloplasty ring has been implanted. The tricuspid repair also makes subsequent ablation of the basal aRV difficult, thus making VT ablation challenging in operated patients. Furthermore, prosthetic tricuspid valve implantation covers the CTI from the RA and makes subsequent trans-catheter access difficult if not impossible. Hence, it is important to have an understanding of not only the clinical arrhythmia and prior surgery but also implications on ablative strategy.

## Pacing

The need for permanent pacing in EA is approximately required in 3.7% of patients, most commonly for AV block, followed by sinus node dysfunction [47]. The anatomical abnormality of the TV, severely dilated RA, and TV repair or replacement may pose a challenge in the placement of pacing leads. Active fixation leads are to be referred to reduce lead displacement especially when there is severe tricuspid regurgitation. Further, the electrophysiologist must be aware of the potential of lead-induced progression of tricuspid regurgitation. The atrialised RV or the true RV can be used for placing the pacing lead, depending on the size of the true RV and the stability obtained. Alternative placement in the coronary sinus tributaries or epicardial leads is a possible option when ventricular pacing lead is necessary. In patients undergoing surgical repair of the TV after placing an endocardial RV pacing lead, the prosthetic ring can be sutured excluding the lead so that the lead crosses outside the ring. Placement of a lead through a bio-prosthetic valve is a feasible option when the need for pacing arises after the surgery [3, 47]. Finally, it must be emphasised that patients with an endocardial lead and an intracardiac shunt have a higher risk of developing thromboembolic event though it is unclear if the routine use of anticoagulants is beneficial in this setting [48].

Conduction system pacing, whether through His bundle pacing (HBP) or left bundle branch area pacing (LBBA), is feasible in EA [49, 50]. His bundle pacing can be challenging due to signals often being observed in the atrialised portion of the ventricles. LBBA pacing, on the other hand, has lower pacing thresholds and higher R wave amplitudes compared to HBP. However, performing LBBA pacing in patients with a dilated right atrium and right ventricle can be difficult. The use of a 9F coronary sinus (CS) left ventricular (LV) lead delivery outer sheath (Medtronic Attain Command MB2; Medtronic Inc.) can aid in providing sufficient backup to the standard A C315 His delivery sheath (Medtronic Inc., Minneapolis, MN). This ensures deep lead fixation in the atrial side of the septum near the septal tricuspid leaflet in the atrialised right ventricle [50].

## Sudden death

EA without any surgical intervention has a reported frequency of sudden death of 16% [11]. The cumulative 70-year mortality in EA is reported to be 14.6%, with an incidence of sudden death being 2 per 1000 patient years. Strong predictors of sudden death include syncope, VT, heart failure, RV dysfunction, and TV surgery [51]. Despite this evidence, there are currently no clear consensus guidelines on selecting patients for offering an ICD for primary prophylaxis. After implementing an aggressive preoperative electrophysiological studies in patients undergoing cone repair, no sudden cardiac deaths were reported on follow-up [44].

## Future directions

Despite advancement in mapping techniques and radiofrequency ablation, recurrence rate after ablation is high. Use of intracardiac echocardiography appears promising in improving outcomes. More is to be known regarding the role of high-density mapping and its utility in improving the ablative success as well as early identification of substrate for ventricular tachycardia especially those arising from the aRV. The current decision-making for surgical repair of the tricuspid valve does not consider the propensity to develop arrhythmias, or potential worsening of arrhythmic substrate. Since dilatation of the cardiac chambers increases the risk of atrial fibrillation and other arrhythmias, it seems prudent to consider right atrial and ventricular volumes and intervene early with surgical repair. Similarly, though preoperative EP study is recommended prior to surgical repair, prophylactic ablation is not currently considered except for accessory pathway. Considering the technical challenges and lesser success rates of cavotricuspid isthmus ablation after tricuspid valve repair/replacement, preoperative ablation may be beneficial. However, these concepts need to be validated with prospective studies. Yet another arena that demands attention is early identification of patients at risk for sudden death. Finally, more is to be known about the optimal surgical techniques for preventing arrhythmias since these may be adopted during surgical repair to prevent/ treat arrhythmias especially atrial fibrillation.

## Conclusions

EA is a complex congenital condition linked to a plethora of arrhythmias whose treatment is challenging. Integration of multimodality imaging with the use of ICE and newer mapping/ablation procedure technologies can improve the success rate and decrease recurrence rates of accessory pathway ablation. Having an in-depth understanding of the cardiac anatomical defects, specific arrhythmia mechanisms and ablative techniques are of utmost importance for an electrophysiologist for better management of these patients.
